# Crystal structures and kinetics of *N*-acetylneuraminate lyase from *Fusobacterium nucleatum*

**DOI:** 10.1107/S2053230X18012992

**Published:** 2018-10-17

**Authors:** Jay Prakash Kumar, Harshvardhan Rao, Vinod Nayak, S. Ramaswamy

**Affiliations:** a Technologies for the Advancement of Science, Institute for Stem Cell Biology and Regenerative Medicine, NCBS, GKVK Campus, Bangalore, Karnataka 560 065, India; b School of Life Science, The University of Trans-Disciplinary Health Sciences and Technology (TDU), Bangalore, Karnataka 560 065, India

**Keywords:** *N*-acetylneuraminate lyase, sialic acid catabolism, enzyme kinetics, *Fusobacterium nucleatum*

## Abstract

*N*-Acetyl-d-neuraminic acid lyase (NanA) catalyzes the breakdown of sialic acid (Neu5Ac) to *N*-acetyl-d-mannosamine (ManNAc) and pyruvate. NanA plays a key role in Neu5Ac catabolism in many pathogenic and bacterial commensals where sialic acid is available as a carbon and nitrogen source. Several pathogens or commensals decorate their surfaces with sialic acids as a strategy to escape host innate immunity. Catabolism of sialic acid is key to a range of host–pathogen interactions. In this study, atomic resolution structures of NanA from *Fusobacterium nucleatum* (FnNanA) in ligand-free and ligand-bound forms are reported at 2.32 and 1.76 Å resolution, respectively*. F. nucleatum* is a Gram-negative pathogen that causes gingival and periodontal diseases in human hosts. Like other bacterial *N*-acetylneuraminate lyases, FnNanA also shares the triosephosphate isomerase (TIM)-barrel fold. As observed in other homologous enzymes, FnNanA forms a tetramer. In order to characterize the structure–function relationship, the steady-state kinetic parameters of the enzyme are also reported.

## Abstract 2

Structures of *N*-acetyl-d-neuraminic acid lyase in the ligand-free form and of a covalent Schiff base adduct are reported. The structure and kinetic data reveal the conserved nature of these proteins among Gram-negative bacteria.

## 1. Introduction   

**Figure 1:**
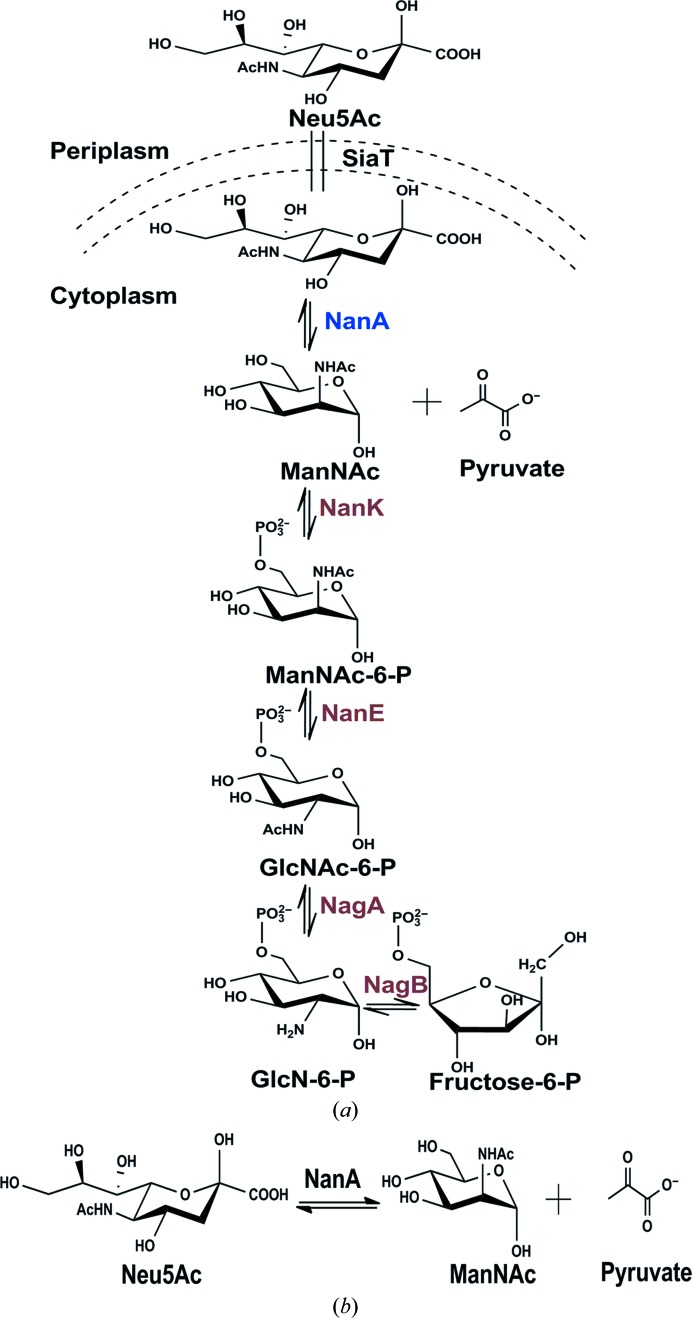
Sialic acid catabolism. (*a*) SiaT, sialic acid transporter; NanA, *N*-acetylneuraminate lyase; NanK, *N*-acetylmannosamine kinase; NanE, *N*-­acetylmannosamine-6-phosphate 2-epimerase; NagA, *N*-acetylglucosamine-6-phosphate deacetylase; NagB, glucosamine-6-phosphate deaminase. (*b*) The reaction catalysed by *N*-acetylneuraminate lyase.

Sialic acids are a family of related nine-carbon, acidic, α-keto sugars which are used by bacteria for molecular mimicry. The most widely recognized type of sialic acid is *N*-acetylneuraminic acid (Neu5Ac; Varki, 1992 ▸; Maru *et al.*, 2002 ▸). The large structural diversity and broad distribution of sialic acids in nature makes them very important in cell biology (Varki & Schauer, 2009 ▸). Sialic acids are usually found as the terminal nonreducing sugars of glycan chains on the cell surface of higher eukaryotes (Huynh *et al.*, 2013 ▸). While some pathogens have evolved *de novo* biosynthesis pathways for Neu5Ac (Vimr *et al.*, 2004 ▸), many bacteria solely rely on the acquisition of Neu5Ac from their environment with the help of specific membrane transporters (Severi *et al.*, 2007 ▸; Bouchet *et al.*, 2003 ▸). Once inside, the sialic acid can either enter the catabolic pathway or the surface-incorporation pathway, depending upon the requirements of the bacteria at the time (North *et al.*, 2016 ▸). The surface-exposed sugar is recognized by the complement system in the serum as ‘self’. Studying the enzymes that are involved in the catabolism of sialic acids is important owing to the biological significance of sialic acids in bacteria. There are five enzymes that are responsible for the catabolism of sialic acids (Fig. 1 ▸ *a*). *N*-Acetylneuraminate lyase (NanA), the first committed enzyme, cleaves Neu5Ac to *N*-acetyl-d-mannosamine (ManNAc) and pyruvate (Fig. 1 ▸ *b*). The second enzyme, *N*-acetylmannosamine kinase (NanK), phos­phorylates ManNAc at the C6 position, which produces *N*-acetyl­mannosamine 6-phosphate (ManNAc-6-P). The third enzyme, *N*-acetylmannosamine-6-phosphate epimerase (NanE), epimerizes ManNAc-6-P to *N*-acetylglucosamine 6-phosphate (GLcNAc-6-P). The fourth enzyme, *N*-acetylglucosamine-6-phosphate deacetylase (NagA), eliminates the acetyl group from GlcNAc-6-P to yield glucosamine 6-phosphate (GlcN-­6-P), which is finally converted to fructose 6-phosphate by the fifth enzyme, glucosamine-6-phosphate deaminase (NagB) (Martinez *et al.*, 1995 ▸; Ringenberg *et al.*, 2003 ▸; Plumbridge & Vimr, 1999 ▸; Vimrt & Troy, 1985 ▸; Fig. 1 ▸ *a*). *N*-Acetyl-d-neuraminic acid lyase (NanA; EC 4.1.3.3) is also known as sialic acid aldolase. NanA is present in both pathogenic and non­pathogenic bacteria and in various mammalian tissues (Izard *et al.*, 1994 ▸; Helmer & Meyer, 1956 ▸; Aisaka *et al.*, 1991 ▸). On the basis of the reaction mechanism, aldolases can be subdivided into classes I and II. NanA belongs to the class I aldolase family, which is characterized by a triosephosphate isomerase (TIM)-barrel fold and by aldol condensation that proceeds through the formation of a Schiff base intermediate between a conserved lysine residue and the substrate pyruvate (Campeotto *et al.*, 2009 ▸). In contrast, class II aldolases differ in the reaction mechanism, in which intermediates are stabilized by a metal cofactor (for example Zn^2+^; Plater *et al.*, 1999 ▸). Multiple class I aldolase structures have been reported, and all of the structures share the same TIM-barrel fold (Wymer *et al.*, 2001 ▸; Theodossis *et al.*, 2004 ▸; Pauluhn *et al.*, 2008 ▸). Various reports have demonstrated that the enzyme is a tetramer in solution (Huynh *et al.*, 2013 ▸; Aisaka *et al.*, 1991 ▸; Krüger *et al.*, 2001 ▸; Barbosa *et al.*, 2000 ▸). Our study enriches the understanding of *N*-acetyl-d-neuraminic acid lyase in members of the oral microbiome such as *Fusobacterium nucleatum*. *F. nucleatum* is a Gram-negative, nonmotile, anaerobic, spindle-shaped and non-spore-forming bacterium (Kolenbrander *et al.*, 2006 ▸). It plays a vital role in the formation of dental plaque biofilms in humans. Here, we report 2.32 and 1.76 Å resolution structures of *N*-acetylneuraminate lyase from *F. nucleatum* in two forms: ligand-free and with ligand bound as a pyruvate Schiff base. The high-resolution structure of the enzyme is compared with those of other known structures of sialic acid aldolases. The atomic resolution structure will provide a pathway for the improvement of prospective antimicrobials. We also report the steady-state enzyme kinetics of this enzyme from *F. nucleatum*.

## 2. Materials and methods   

### 2.1. Production of *F. nucleatum* NanA   

The gene encoding *F. nucleatum* NanA (FnNanA) was cloned into a pET300/NT-DEST vector as described previously (Bairy *et al.*, 2018 ▸) and transformed into *Escherichia coli* BL21 (DE3) cells (Novagen) for protein expression (Table 1 ▸). The cell cultures were grown to an OD_600 nm_ of 0.6 and induced with 100 µ*M* isopropyl β-d-1-thiogalactopyranoside (IPTG). The cells were grown at 289 K for 16 h post-induction. The cultures were harvested by centrifugation at 6000*g* for 15 min at 277 K. The cell pellets were resuspended in lysis buffer [50 m*M* Tris–HCl pH 8.0, 500 m*M* NaCl, 20 m*M* imidazole and one tablet of cOmplete EDTA-free Protease Inhibitor Cocktail (Roche)]. The cells were lysed by passage through a cell disruptor (Constant Systems) three times at 138 MPa. The cell lysate was centrifuged at 18 000*g* for 35 min to remove cell debris and unlysed cells.

The supernatant was loaded onto a 5 ml HisTrap FF column (GE Healthcare) equilibrated with buffer *A* (50 m*M* HEPES pH 7.4, 20 m*M* imidazole, 500 m*M* NaCl, 6% glycerol, 10 m*M* β-mercaptoethanol) using an ÄKTA FPLC system (GE Healthcare). Unbound bacterial proteins were eluted with a step gradient of 4 and 6% buffer *B* (50 m*M* HEPES pH 7.4, 500 m*M* imidazole, 500 m*M* NaCl, 6% glycerol, 10 m*M* β-mercaptoethanol). The desired protein was eluted using a linear gradient of 6–100% buffer *B*. The fractions were analyzed by SDS–PAGE to check their purity. Prior to cation-exchange chromatography, the protein was buffer-exchanged with 50 m*M* HEPES pH 6.8, 10 m*M* NaCl, 6% glycerol, 10 m*M* β-mercaptoethanol using Amicon Ultra Centrifugal Filters (10 000 molecular-weight cutoff; Millipore). The protein was loaded onto a cation exchanger (5 ml HiTrap SP FF column; GE Healthcare) equilibrated with the same buffer.

The protein was further purified on a Superdex 200 10/300 GL analytical column in 50 m*M* HEPES pH 7.4, 50 m*M* NaCl, 10 m*M* β-mercaptoethanol. The purity of FnNanA was analyzed by 12% SDS–PAGE. The peak fraction with the highest purity was pooled and then concentrated using Amicon Ultra Centrifugal Filters (10 000 molecular-weight cutoff; Millipore) for crystallization trials. All protein-purification steps were performed at 277 K. The protein concentration was determined using the Bradford assay (Bio-Rad) with bovine serum albumin as a protein standard. The absorbance of each sample was measured at 595 nm using an Ultrospec 2100 pro UV–visible spectrophotometer (GE Healthcare). The purified protein exhibited a molecular mass of ∼35 kDa using mass spectrometry, matching the calculated mass of the translated His_6_-tagged protein (34.9 kDa; data not shown).

### 2.2. Crystallization   

**Figure 2:**
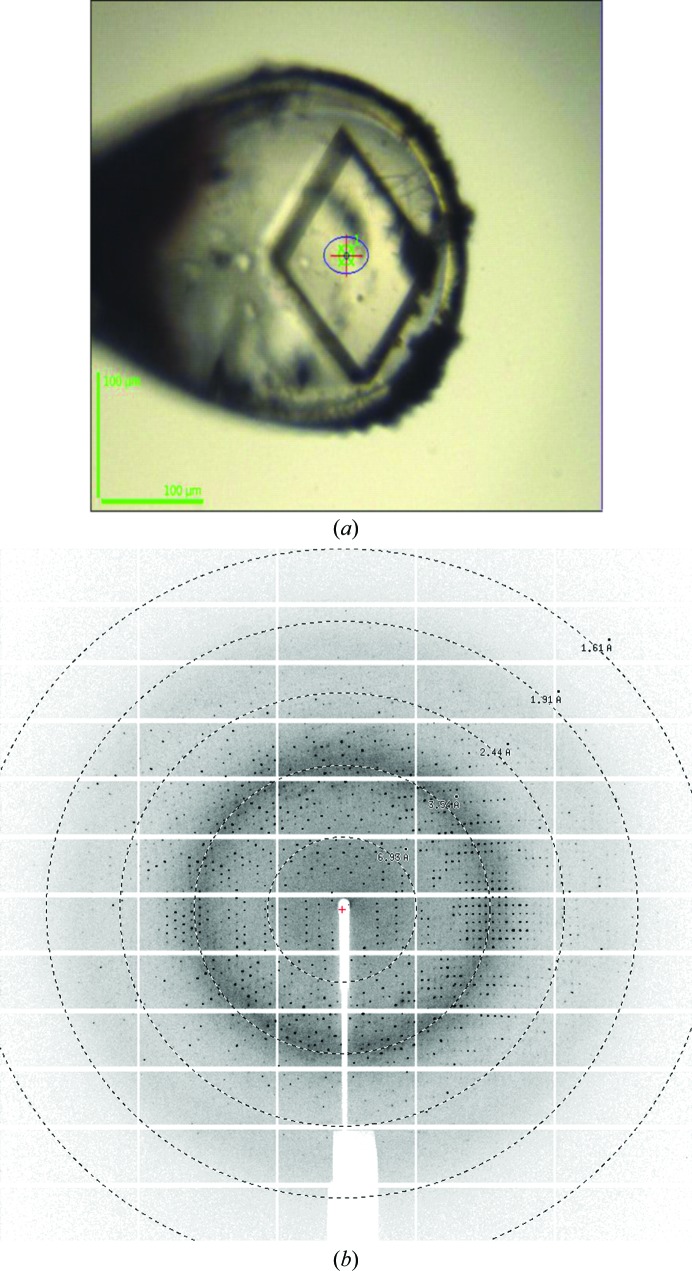
Crystal and diffraction pattern. (*a*) A typical crystal of ligand-free FnNanA. (*b*) X-ray diffraction image of a ligand-free FnNanA crystal showing diffraction to 2.32 Å resolution.

Purified FnNanA was concentrated to 10 mg ml^−1^ in 50 m*M* HEPES pH 7.4, 50 m*M* NaCl, 10 m*M* β-mercaptoethanol for crystallization setup. Initial hanging-drop crystallization trials were performed using commercially available sparse-matrix crystallization screens from Rigaku Reagents (Wizard 1 and 2), Hampton Research (PEG/Ion, PEG/Ion 2, Crystal Screen and Crystal Screen 2) and Qiagen (The Classics and Classics II Suites) using a Mosquito nanolitre-dispensing robot (TTP Labtech). The crystallization drops consisted of 350 nl protein solution and 350 nl reservoir solution. Several of the commercial screen conditions produced small crystals at 277 and 291 K. These initial hits were optimized to obtain diffraction-quality crystals. For the crystallization of FnNanA with sodium pyruvate, a tenfold molar excess of sodium pyruvate was mixed with 10 mg ml^−1^ (0.28 m*M*) protein and incubated for 30 min before crystallization setup. Crystals were obtained after 7–8 d (Fig. 2 ▸ *a*). All crystals were cryoprotected using 20%(*v*/*v*) ethylene glycol in reservoir solution. The crystals were flash-cooled in liquid nitrogen prior to diffraction data collection. Crystallization information is summarized in Table 2 ▸.

### 2.3. Data collection and processing   

X-ray diffraction data were collected from a single crystal of ligand-free FnNanA on beamline ID23 at the European Synchrotron Radiation Facility (ESRF), Grenoble, France at a wavelength of 0.9789 Å. X-ray data for ligand-bound FnNanA with a pyruvate Schiff base were also collected on beamline ID30 at the ESRF at a wavelength of 0.9762 Å. Diffraction data collection was performed at 100 K.

The diffraction data were indexed and integrated using *XDS* (Kabsch, 2010 ▸). Data reduction and scaling were achieved with *AIMLESS* (Evans & Murshudov, 2013 ▸) from the *CCP*4 suite (Winn *et al.*, 2011 ▸). The resulting intensity data were analyzed using *phenix.xtriage* from the *PHENIX* suite (Zwart *et al.*, 2005 ▸; Adams *et al.*, 2010 ▸). An estimate of the number of molecules in the asymmetric unit was obtained using *MATTHEWS_COEF* from the *CCP*4 suite (Matthews, 1968 ▸; Kantardjieff & Rupp, 2003 ▸). A summary of the data-collection and processing statistics is given in Table 3 ▸.

### 2.4. Structure solution and refinement   

A monomer of *Pasteurella multocida* NanA (PDB entry 4imc; Huynh *et al.*, 2013 ▸), which has 73% sequence identity to FnNanA, was used as a search model for molecular replacement with *Phaser* (McCoy *et al.*, 2007 ▸) in the *PHENIX* suite for both ligand-free FnNanA and ligand-bound FnNanA with a pyruvate Schiff base. The *phenix.autobuild* program (Terwilliger *et al.*, 2008 ▸) was used for initial model building. Structure refinement was performed using *phenix.refine* (Afonine *et al.*, 2012 ▸). Iterative improvement of the map and the model was performed using alternate cycles of refinement and residue-by-residue analysis in *Coot* (Emsley *et al.*, 2010 ▸). Water and ligand molecules were added *via Coot* and modelled into the electron density manually. All structure-refinement statistics are summarized in Table 4 ▸.

### 2.5. Sequence comparison based on the structural alignment   

The *T-Coffee Expresso* server (Armougom *et al.*, 2006 ▸) was used to produce a structure-based sequence alignment of the four known *N*-acetylneuraminate lyase structures, and a figure was generated with *ESPript* 3 (Robert & Gouet, 2014 ▸). The sequence identities of the *N*-acetylneuraminate lyases from *Haemophilus influenzae* (PDB entry 1f7b; Barbosa *et al.*, 2000 ▸), *P. multocida* (PDB entry 4imd; Huynh *et al.*, 2013 ▸), *Staphylococcus aureus* (PDB entry 5a8g; Stockwell *et al.*, 2016 ▸) and *E. coli* (PDB entry 1nal; Izard *et al.*, 1994 ▸) are 74, 72, 57 and 36%, respectively.

### 2.6. Enzyme assay   

*N*-Acetylneuraminate lyase activity was assessed by quantifying the amount of pyruvate produced using a standard coupled assay (Zhu *et al.*, 2010 ▸). The pyruvate released by the cleavage of *N*-acetylneuraminic acid is oxidized by pyruvate oxidase in the presence of phosphate (P_i_) and oxygen to give acetylphosphate, CO_2_ and H_2_O_2_. Horseradish peroxide (HRP) was used to catalyze the reaction between the fluorometric probe and H_2_O_2_. The resulting H_2_O_2_ was then detected using a fluorogenic substrate (Sugahara *et al.*, 1980 ▸).

## 3. Results and discussion   

### 3.1. Structure determination   

The structure of ligand-free FnNanA was refined to 2.32 Å resolution. The structure has 0.35% Ramachandran outliers. The conserved Ser79 residue is in the disallowed region of the Ramachandran plot in each monomer of ligand-free FnNanA. However, electron density is well defined for Ser79.

The crystals of ligand-bound FnNanA with a pyruvate Schiff base intermediate belonged to space group *P*2_1_2_1_2 and diffracted to 1.76 Å resolution. The two monomers that were identified appear to form a tetramer with a similar monomer arrangement to those in sialic acid aldolases from other species. The structure has 0.17% Ramachandran outliers. One residue, Lys267 of chain *B*, is present in the disallowed region; the electron density for this residue is not well defined. All residues were modelled into electron density, except for Tyr108 and Lys109 of chain *B* in ligand-bound FnNanA with a pyruvate Schiff base and the residues at the N-termini of all monomers.

### 3.2. The overall structure of *N*-acetylneuraminate lyase   

**Figure 3:**
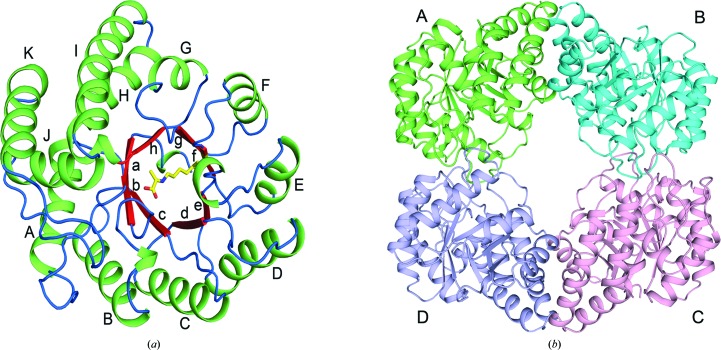
The overall crystal structure of *N*-acetylneuraminate lyase from *F. nucleatum*. (*a*) Cartoon representation of FnNanA with individually coloured secondary structures. Secondary-structure elements are labelled sequentially (with lower case letters corresponding to β-strands and upper case letters corresponding to α-helices). The Lys161–pyruvate Schiff base intermediate in the active site is displayed as sticks with yellow C atoms. (*b*) The tertiary structure (tetramer) of FnNanA with each subunit shown in cartoon presentation in a different colour. The figures were generated using the *PyMOL* molecular-graphics system (DeLano, 2002 ▸).

The monomeric structure of FnNanA shows that the enzyme adopts a classical TIM (β/α)_8_-barrel tertiary-structure fold (Fig. 3 ▸ *a*). In addition to the eight helices that make up the TIM barrel, FnNanA has three additional α-helices (I, J and K) at the C-terminus (Fig. 3 ▸ *a*). The NanAs from *H. influenzae* (Barbosa *et al.*, 2000 ▸) and *P. multocida* (Huynh *et al.*, 2013 ▸) also have these three additional helices. We compared the structures of ligand-free FnNanA and ligand-bound FnNanA with a pyruvate Schiff base. The overall architecture of the ligand-bound FnNanA with a pyruvate Schiff base is similar to that of ligand-free FnNanA. There are no major structural changes in FnNanA upon the binding of pyruvate. All four subunits in ligand-free FnNanA are structurally similar, and superpose with a root-mean-square deviation (r.m.s.d.) of between 0.127 and 0.169 Å for 290 Cα atoms. The monomer of FnNanA bound to pyruvate superposes with an r.m.s.d. 0.836 Å onto monomers from the same asymmetric unit.

The Cα atoms of chain *A* of the ligand-free *N*-acetylneuraminate lyases from *F. nucleatum*, *H. influenzae* (PDB entry 1f5z; Barbosa *et al.*, 2000 ▸) and *P. multocida* (PDB entry 4imc; Huynh *et al.*, 2013 ▸) superpose with r.m.s.d.s of 1.12 and 1.14 Å, respectively. The Cα atoms of chain *A* of the ligand-bound *N*-acetylneuraminate with a pyruvate Schiff base from *F. nucleatum*, *P. multocida* (PDB entry 4imd; Huynh *et al.*, 2013 ▸) and *S. aureus* (PDB entry 4ah7; Timms *et al.*, 2013 ▸) superposed with r.m.s.d.s of 0.54 and 0.78 Å, respectively. The quaternary structure of the *N*-acetylneuraminate lyase (PDB entry 5zjm) was analyzed and assembled into a tetramer (Fig. 3 ▸ *b*) using the *PISA* server (Krissinel & Henrick, 2007 ▸). All four crystallographically independent monomers are very similar. Small-angle X-ray scattering data of the soluble protein also concurred that the protein was a tetramer (data not shown).

**Figure 4:**
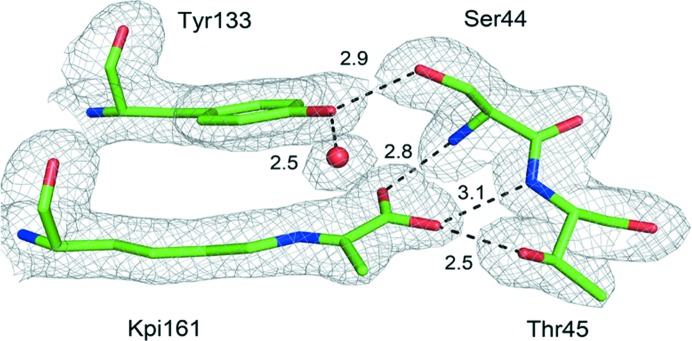
Electron-density map of the Schiff base formed by Lys161 with pyruvate. Complex showing the interaction of the pyruvate enamine with the enzyme. The pyruvate is covalently linked to Lys161 and makes hydrogen bonds to Ser44 and Thr45. The hydrogen-bonding network to Tyr133 and a conserved water molecule are also shown. The electron density represented by the grey mesh was contoured at the 1σ level, showing continuous density linking the C2 atom of pyruvate and the N∊ atom of Lys161. This figure was generated using the *PyMOL* molecular-graphics system (DeLano, 2002 ▸).

The observed electron-density map for ligand-bound FnNanA with a pyruvate Schiff base in chain *A* clearly showed that the N∊ atom of Lys161 is conjugated as a Schiff base to the C2 atom of pyruvate. The carboxylate group of the pyruvate makes hydrogen bonds to the backbone N atom of Ser44 and to the hydroxyl group of Thr45. In addition, the Tyr133 side chain makes a hydrogen bond to the hydroxyl group of Ser44 and a conserved water molecule (Fig. 4 ▸). Tyr133 lies almost parallel to the Schiff base intermediate and is engaged in hydrogen bonding for the Schiff base reaction. The atomic coordinates have been submitted to the Protein Data Bank (PDB) and the PDB codes for ligand-free FnNanA and ligand-bound FnNanA with a pyruvate Schiff base are 5zjm and 5zka, respectively.

### 3.3. Sequence comparison based on structural alignment   

**Figure 5:**
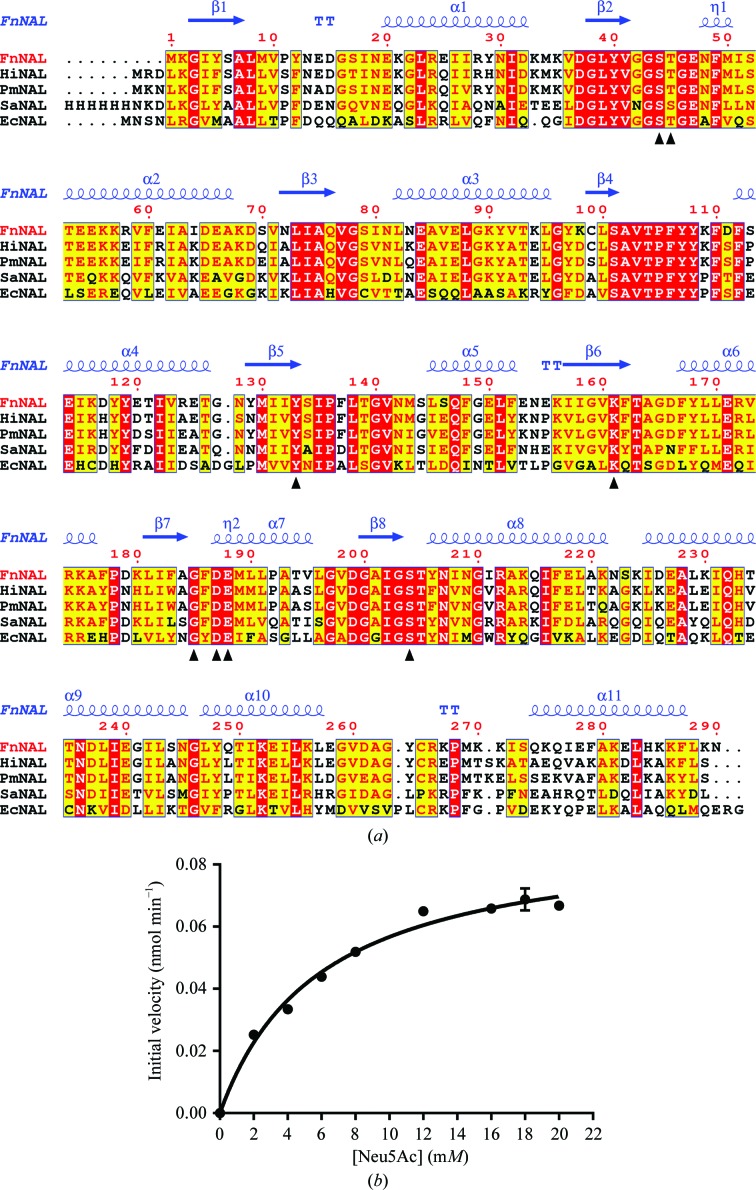
Structure-based alignment of *F. nucleatum* *N*-acetylneuraminate lyase (FnNAL) and selected *N*-acetylneuraminate lyases (NALs) of known structure. (*a*) The sequence identities to the *N*-acetylneuraminate lyases from *H. influenzae* (HiNAL; PDB entry 1f7b; Barbosa *et al.*, 2000 ▸), *P. multocida* (PmNAL; PDB entry 4imd; Huynh *et al.*, 2013 ▸), *S. aureus* (SaNAL; PDB entry 5a8g; Stockwell *et al.*, 2016 ▸) and *E. coli* (EcNAL; PDB entry 1nal; Izard *et al.*, 1994 ▸) are 74, 72, 57 and 36%, respectively. The structural alignment was computed using the *T-Coffee Expresso* server (Armougom *et al.*, 2006 ▸) and the figure was produced with *ESPript* 3 (Robert & Gouet, 2014 ▸). Strictly conserved residues across NAL enzymes are shown on a red background. The secondary structure of FnNanA is shown at the top; coils represent helices and arrows represent β-strands. The residues involved in the active site are labelled with small black triangles. (*b*) Kinetic analysis of *F. nucleatum* *N*-acetylneuraminate lyase with Neu5Ac. The data were fitted to the Michaelis–Menten equation with an *R* ^2^ value of 0.99.

Structure-based sequence alignment of FnNanA and four known *N*-acetylneuraminate lyase structures was carried out using the *T-Coffee Expresso* server (Fig. 5 ▸ *a*; Armougom *et al.*, 2006 ▸). Sequence alignment showed that FnNanA contains conserved catalytic sites (Lys161 and Tyr133) and the conserved specific substrate (Neu5Ac) binding motif G*XX*GE (Barbosa *et al.*, 2000 ▸; Ji *et al.*, 2015 ▸; García García *et al.*, 2012 ▸). The G*XX*GE motif, which is situated between positions 43 and 47 (FnNanA numbering), is involved in substrate recognition, and the *XX* residues are usually S and/or T. Apart from this, a group of amino acids (Asp187, Glu188 and Ser204) are also involved in binding the carbohydrate moiety.

### 3.4. Enzyme assay   

The data were fitted to the Michaelis–Menten kinetic model (Fig. 5 ▸ *b*). The Michaelis–Menten constant (*K*_m_) was found to be 6.0 ± 0.6 m*M*, *V*_max_ was calculated to be 0.090 ± 0.003 nmol min^−1^ and *k*_cat_ was determined to be 12.5 ± 0.5 s^−1^. A comparison with the Michaelis–Menten kinetic constants of related NanA enzymes reveals that the kinetic characteristics of this enzyme (both *k*_cat_ and *K*_m_) are quite similar to those reported in the literature (Uchida *et al.*, 1984 ▸; North *et al.*, 2016 ▸; Table 5 ▸). We report the kinetic analysis of *N*-acetyl­neuraminate lyase from *F. nucleatum*.

## 4. Conclusion   

In this paper, we present the crystal structures of ligand-free FnNanA and ligand-bound FnNanA with a pyruvate Schiff base. Analysis and comparison of the structures show a conserved TIM-barrel fold. Enzyme kinetics were determined and were observed to be similar to those of orthologs from other species. Atomic resolution structures of *N*-acetylneuraminate lyases from pathogenic bacteria such *F. nucleatum* will provide us with the structural information necessary for future antimicrobial development.

## Supplementary Material

PDB reference: *N*-acetylneuraminate lyase from *F. nucleatum*, 5zjm

PDB reference: pyruvate Schiff-base intermediate, 5zka

## Figures and Tables

**Table 1:** *F. nucleatum* NanA production information

Source organism	*F. nucleatum* strain ATCC 25586
DNA source	Synthetic DNA
Forward primer	CAAAAAAGCAGGCTTCATGAAAGGGATATATTCAG
Reverse primer	CAAGAAAGCTGGGTTTTAATTTTTTAAAAATTTTTTATG
Cloning vector	pMK vector
Expression vector	pET300/NT-DEST with an N-terminal His tag
Expression host	*E. coli* BL21 (DE3)
Sequence of the recombinant protein produced†	MHHHHHHITSLYKKAGFMKGIYSALMVPYNEDGSINEKGLREIIRYNIDKMKVDGLYVGGSTGENFMISTEEKKRVFEIAIDEAKDSVNLIAQVGSINLNEAVELGKYVTKLGYKCLSAVTPFYYKFDFSEIKDYYETIVRETGNYMIIYSIPFLTGVNMSLSQFGELFENEKIIGVKFTAGDFYLLERVRKAFPDKLIFAGFDEMLLPATVLGVDGAIGSTYNINGIRAKQIFELAKNSKIDEALKIQHTTNDLIEGILSNGLYQTIKEILKLEGVDAGYCRKPMKKISQKQIEFAKELHKKFLKN

† Non-native amino-acid residues originating from the vector are underlined.

**Table 2:** Crystallization of *F. nucleatum* NanA

	Ligand-free FnNanA	Ligand-bound FnNanA with a pyruvate Schiff base
Method	Vapour diffusion, hanging drop	Vapour diffusion, hanging drop
Plate type	96-well	96-well
Temperature (K)	277	277
Protein concentration (mg ml^−1^)	10	10
Buffer composition of protein solution	50 m*M* HEPES pH 6.8, 50 m*M* NaCl, 10 m*M* β-mercaptoethanol	50 m*M* HEPES pH 6.8, 50 m*M* NaCl, 10 m*M* β-mercaptoethanol
Buffer composition of reservoir solution	0.1 *M* CHES pH 9.5, 10%(*w*/*v*) PEG 3000, 5 m*M* adenosine 5′-triphosphate disodium salt hydrate (ATP)	0.1 *M* CHES pH 9.5, 10%(*w*/*v*) PEG 3000, 2.85 m*M* sodium pyruvate
Volume of drop (nl)	700	700
Volume of reservoir (µl)	100	100

**Table 3:** Data collection and processing for *F. nucleatum* NanA Values in parentheses are for the outer shell.

	Ligand-free FnNanA	Ligand-bound FnNanA with a pyruvate Schiff base
Diffraction source	Beamline ID23-1, ESRF	Beamline ID30B, ESRF
Wavelength (Å)	0.9789	0.9762
Temperature (K)	100	100
Detector	Dectris PILATUS 6M-F	Dectris PILATUS3 6M
Crystal-to-detector distance (mm)	405.721	318.80
Rotation range per image (°)	0.1	0.05
Total rotation range (°)	180	230
Exposure time per image (s)	0.1	0.01
Space group	*C*2	*P*2_1_2_1_2
*a*, *b*, *c* (Å)	105.17, 108.98, 141.14	82.68, 86.57, 89.99
α, β, γ (°)	90, 96.9, 90	90, 90, 90
Mosaicity (°)	0.14	0.13
Resolution range (Å)	49.24–2.32 (2.38–2.32)	86.57–1.76 (1.79–1.76)
Total No. of reflections	150647 (9709)	213363 (12153)
No. of unique reflections	64567 (4304)	63236 (3548)
Completeness (%)	94.900 (94.300)	98.000 (98.200)
Multiplicity	2.300 (2.300)	3.400 (3.400)
〈*I*/σ(*I*)〉	10.300 (1.7)†	10.900 (1.6)‡
*R*_r.i.m._	0.051 (0.600)	0.064 (0.999)
*R*_p.i.m._	0.032 (0.371)	0.034 (0.520)
Overall *B* factor from Wilson plot (Å^2^)	59.610	29.190

† We decided to cut the data at 〈*I*/σ(*I*)〉 = 2.32 Å as the CC_1/2_ and multiplicity of the data in the outer shell were 0.88 and 2.3, respectively. 〈*I*/σ(*I*)〉 falls below 2.0 in the outer shell at 2.38 Å resolution.

‡ We decided to cut the data at 〈*I*/σ(*I*)〉 = 1.76 Å as the CC_1/2_ and multiplicity of data in the outer shell were 0.65 and 3.4, respectively. 〈*I*/σ(*I*)〉 falls below 2.0 in the outer shell at 1.82 Å resolution.

**Table 4:** Structure solution and refinement of *F. nucleatum* NanA Values in parentheses are for the outer shell.

	Ligand-free FnNanA	Ligand-bound FnNanA with a pyruvate Schiff base
Resolution range (Å)	47.10–2.32 (2.35–2.32)	60.88–1.76 (1.80–1.76)
Completeness (%)	94.5	97.7
σ Cutoff	1.3	1.3
No. of reflections		
Working set	61188 (2602)	61155 (4313)
Test set	3195 (141)	2028 (147)
Final *R*_cryst_	0.205 (0.4388)	0.187 (0.3258)
Final *R*_free_	0.243 (0.4569)	0.216 (0.3769)
No. of non-H atoms		
Protein	8895	4618
Ligand	12	30
Solvent	14	166
Total	8921	4814
R.m.s. deviations		
Bonds (Å)	0.004	0.007
Angles (°)	1.01	1.12
Average *B* factors (Å^2^)		
Overall	68.03	38.71
Protein	68.02	38.55
Ligand	82.75	60.89
Ramachandran plot		
Most favoured (%)	97.57	97.23
Allowed (%)	2.08	2.6
No. of TLS groups	4	2

**Table 5:** Kinetic parameters of FnNanA and previously characterized *N*-acetylneuraminate lyases from *E. coli* (EcNanA), *P. multocida* (PmNanA) and methicillin-resistant *S. aureus* (MRSA NanA)

Enzymes	*K*_m_ (m*M*)	*k*_cat_ (s^−1^)	*k*_cat_/*K*_m_ (s^−1^ m*M* ^−1^)
FnNanA†	6.0 ± 0.6	12.5 ± 0.5	2.1
EcNanA‡	2.5 ± 0.3	10 ± 0.4	4
PmNanA‡	4.9 ± 0.7	16 ± 1	3
MRSA NanA§	3.2 ± 0.1	22 ± 0.1	6.9

† Current study.

‡ Data taken from Uchida *et al.* (1984 ▸).

§ Data taken from North *et al.* (2016 ▸).
